# Unveiling the Therapeutic Potential of Targeting RRM2 in Hepatocellular Carcinoma: An Integrated In Silico and In Vitro Study

**DOI:** 10.1007/s10142-025-01630-0

**Published:** 2025-06-10

**Authors:** Lobna Ibrahim, Rania Hassan Mohamed, Mahmoud M. Tolba, Sara M. Radwan, Nadia M. Hamdy, Mahmoud Elhefnawi

**Affiliations:** 1 Biochemistry Department, Faculty of Pharmacy, Ain Shams University, Abassia, Cairo, 11566 Egypt; 2 Biochemistry Department, Faculty of Science, Ain Shams University, Abassia, Cairo, 11566 Egypt; 3 Clinical Research and Pharmaceutical Division, Ministry of Health and Population, Cairo, Egypt; 4 Biomedical Informatics and Chemoinformatics Group, Informatics and Systems Department, National Research Centre, Cairo, Egypt

**Keywords:** Hepatocellular carcinoma, Liver cancer, Bioinformatic analysis, RRM2, CRISPR/Cas9

## Abstract

Hepatocellular carcinoma (HCC) has attracted global and local interest, with alarming current and forecasted statistics. This is mainly due to its late-stage diagnosis, high recurrence rate and potential resistance to conventional therapies. Thus, there is an emerging need to explore new molecular targets and novel treatment strategies to effectively combat HCC. In this study, we analyzed four GEO datasets (GSE112790, GSE62232, GSE60502 and GSE84402) to identify upregulated differentially expressed genes (DEGs) associated with HCC. Enrichment analysis and protein‒protein interaction (PPI) network construction were performed on the overlapping upregulated DEGs to predict and prioritize potential therapeutic targets. Six hub genes (RRM2, TOP2A, CCNB1, CDK1, BIRC5 and PBK) were identified, with RRM2 emerging as the top candidate. The role of RRM2 in HCC was then validated through a literature review, in silico analysis and CRISPR/Cas9-mediated gene disruption, followed by in vitro functional assays in HepG2 cells. RRM2 knockdown resulted in significant reductions in cell viability, proliferation inhibition, migration impairment, G2/M phase cell cycle arrest and apoptosis. Our findings emphasize RRM2 as a critical player in HCC progression. Targeting RRM2 with CRISPR/Cas9 effectively reversed several hallmarks of HCC, offering new opportunities for the development of precise HCC therapeutic options.

**Graphical Abstract:**

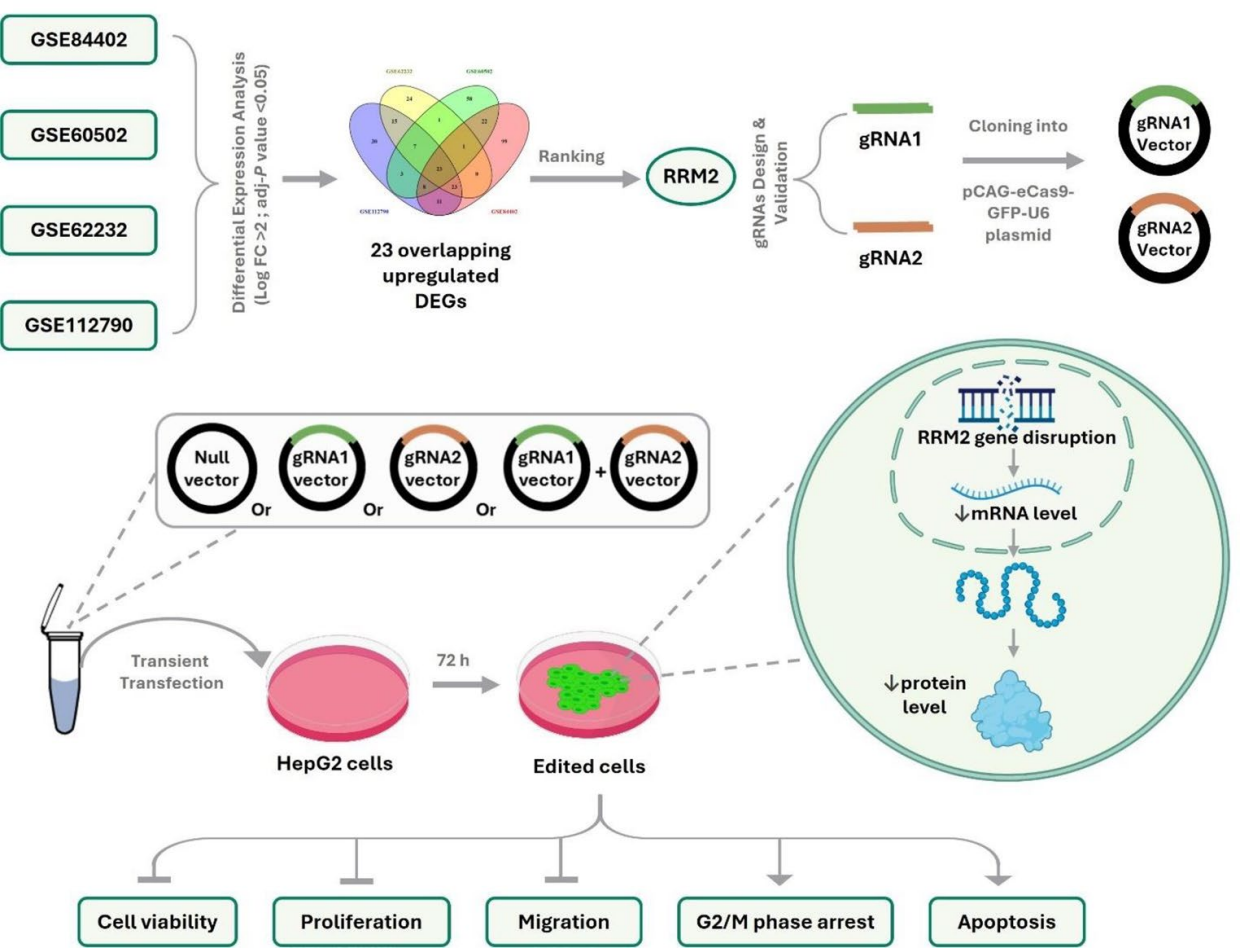

**Supplementary Information:**

The online version contains supplementary material available at 10.1007/s10142-025-01630-0.

## Background

Liver cancer is a major global health concern, accounting for approximately 758,725 deaths annually (Bray et al. 2024; Ferlay J, 2024). It ranks as the third leading cause of cancer mortality and the sixth most frequently diagnosed cancer, with 866,136 new cases recorded annually (Ferlay J, 2024). In Egypt, liver cancer is particularly challenging, mainly because of its economic and social burdens. It has the highest incidence and mortality rates of all cancer types, with forecasts suggesting a 126% increase in new cases and deaths by 2050 (erlay J, 2024).

Hepatocellular carcinoma (HCC), the most common type of primary liver cancer, represents 75-90% of liver cancer cases (Bray et al. 2024; Chakraborty and Sarkar 2022). The etiology of HCC is multifactorial, with chronic viral infection (HCV and HBV) being the most prominent risk factor responsible for 21–55% of HCC cases globally (Bray et al. 2024; El-Mesallamy et al. 2011). This is due to their tendency to cause persistent liver inflammation, fibrosis, and cirrhosis, which are precursors to malignant transformation (Feng and Zhao 2024; Hammad et al. 2022, 2023). Moreover, metabolic factors, including nonalcoholic fatty liver disease (NAFLD) and nonalcoholic steatohepatitis (NASH), have emerged as significant contributors to the increasing incidence of HCC, particularly in the context of rising global obesity and diabetes rates (Chidambaranathan-Reghupaty et al. 2021; Omar et al. 2023). Additional risk factors include aflatoxin exposure, heavy alcohol consumption, genetic and immune-related disorders, and demographic factors (Bray et al. 2024; Chidambaranathan-Reghupaty et al. 2021; Feng and Zhao 2024).

Traditional treatment options for HCC, including surgical resection, liver transplantation, transarterial chemoembolization, and radiotherapy, are often associated with limited efficiency, which leads to high recurrence rates and therapeutic resistance (Chakraborty and Sarkar 2022; Feng and Zhao 2024). This is mainly due to the diagnosis of liver cancer in advanced stages, as it is usually asymptomatic in early stages (Chakraborty and Sarkar 2022). Systemic treatments, such as tyrosine kinase inhibitors, have shown some efficacy in extending patient survival; however, their overall impact remains modest (Chidambaranathan-Reghupaty et al. 2021). Given these limitations, combined with the clinical and molecular heterogeneity of HCC, there is a growing need to explore new molecular targets and employ advanced therapeutic approaches (Hamdy et al. 2024), such as gene- or immune-based therapies (Elanany et al. 2023) or combination therapy (Atta et al.), with the hope of improving treatment outcomes and enhancing both overall and recurrence-free survival (Chakraborty and Sarkar 2022; Singal et al. 2023).

The increasing availability of multiomics data in public databases, together with recent advances in bioinformatic tools, offers a powerful approach for predicting potential data-driven targets involved in HCC pathogenesis (Jimenez-Santos et al. 2022; Tsimberidou et al. 2022; Zhang et al. 2022). Moreover, clustered regularly interspaced short palindromic repeats (CRISPR)/CRISPR-associated protein 9 (Cas9) is a breakthrough gene-editing tool that has shown promising outcomes in several preclinical studies and has demonstrated great potential in targeting various genetic disorders and cancers, including HCC (Amjad et al. 2024; Liu et al. 2024; Wu et al. 2020). Thus, the use of the CRISPR/Cas9 technique represents a promising treatment strategy by precisely targeting and disrupting predicted upregulated genes that are involved in HCC (“18th International Conference of Biochemistry & Molecular Biology (18th ICBMB),” 2023).

In the present study, we aimed to bridge the gap between predicting potential targets and validating them experimentally. Through bioinformatic analysis, we identified promising hub genes involved in HCC pathogenesis and selected the most promising candidate gene for experimental validation. Hence, we employed the CRISPR/Cas9 technique to precisely knock down the predicted target gene and explored the therapeutic potential of inhibiting this gene in vitro in HepG2 cells. This integrated approach, which combines bioinformatics, gene editing, and in vitro experiments, represents a crucial step toward personalized and precise treatment options for HCC, addressing the urgent need for new therapeutic targets and strategies to overcome this challenging disease.

## Methods

### Bioinformatic analysis for the prediction of potential upregulated targets in HCC

Four independent microarray gene expression datasets (“GSE112790”, “GSE62232”, “GSE60502” and “GSE84402”) (Schulze et al. 2015; Shimada et al. 2019; Wang et al. 2014, 2017) were retrieved from the Gene Expression Omnibus database (GEO; https://www.ncbi.nlm.nih.gov/geo) (Barrett et al. 2013). These datasets were selected on the basis of defined criteria to ensure biological relevance and technical robustness, including studies of untreated human HCC tissue with clear tumor versus non-tumor comparisons (either paired or unpaired) and excluding datasets involving other cancer types, combined tumors, or experimental interventions to ensure that the observed expression changes reflect disease-specific changes rather than treatment effects. The detailed information for these datasets is summarized in Table 1. The GEO2R tool (http://www.ncbi.nlm.nih.gov/geo/geo2r) was subsequently used to analyze each dataset separately to identify the upregulated differentially expressed genes (DEGs) between non-tumor and tumor liver tissue samples, with a log fold change (FC) ≥ 2 and an adjusted *P*-value (adj.*P*-value) < 0.05 were set as thresholds based on both statistical considerations and a literature review (Shi et al. 2008; Shimada et al. 2019).

The upregulated DEGs were intersected and visualized via the Venny tool (v 2.1, https://bioinfogp.cnb.csic.es/tools/venny/) (Oliveros, 2007–2015). Furthermore, the Database for Annotation, Visualization, and Integrated Discovery (DAVID 2021, https://david.ncifcrf.gov/) (Huang da et al. 2009; Sherman et al. 2022) was used to perform enrichment analysis for the overlapping upregulated DEGs, including gene ontology (GO) terms and Kyoto Encyclopedia of Genes and Genomes (KEGG) pathway analysis, and then the output was visualized through the SRplot (https://www.bioinformatics.com.cn/srplot) web tool (Tang et al. 2023). A *P*-value < 0.05 was considered significant.

A PPI network was constructed for these overlapping upregulated DEGs using the STRING (v 12, https://string-db.org/) tool (Szklarczyk et al. 2023), with a minimum interaction score threshold of 0.7 (high confidence). Then, the network was analyzed with Cytoscape software (v3.10.2) (Shannon et al. 2003) and the CytoHubba plugin (v 0.1) (Chin et al. 2014) to identify the top hub genes according to the following metrics: maximum clique centrality (MCC), maximum neighborhood component (MNC), degree of connectivity, and bottleNeck. The combined score for each gene was calculated by averaging the normalized values of these four metrics (MCC, MNC, degree of connectivity and bottleNeck) via the min–max normalization method.

**Table 1:** Detailed information on the four microarray datasets used in this study.

GEO ID	Description	Tumor	Non-Tumor	Platform	Country	Type
GSE112790	HCC liver tumor samples vs. normal liver samples	181	15	GPL570	Japan	Expression profiling by array for *Homo Sapiens* species.
GSE62232	HCC liver tumor samples vs. normal liver samples	81	10	GPL570	France	
GSE60502	HCC liver tissue samples vs. adjacent non-tumor tissues	18	18	GPL96	Taiwan	
GSE84402	HCC liver tissue samples vs. adjacent non-tumor tissues	14	14	GPL570	China	

**GEO**:Gene Expression Omnibus, **GSE**: GEO series, **GPL**: GEO platform, **HCC**: hepatocellular carcinoma. Data were retrieved from the GEO database (https://www.ncbi.nlm.nih.gov/geo), accessed on [August 2022], and revised on [March 2024]

### In Silico exploration of the role of RRM2 in HCC

A literature review and in silico analysis were performed to explore the role of the top predicted candidate (RRM2) in HCC. Gene expression profiling interactive analysis (GEPIA2, http://gepia2.cancer-pku.cn/) (Tang et al. 2019) was used to analyze RRM2 mRNA expression in the Liver Hepatocellular Carcinoma (LIHC) dataset compared with that in normal liver tissues. The associations between RRM2 expression and LIHC tumor grade and stage were investigated through the University of ALabama at Birmingham CANcer data analysis Portal (UALCAN, https://ualcan.path.uab.edu/analysis-prot.html) (Chandrashekar et al. 2017, 2022). Moreover, the Kaplan–Meier plotter (http://kmplot.com/analysis/) (Menyhárt et al. 2018) was used to investigate the impact of RRM2 expression on liver cancer patients’ overall survival (OS). Additionally, the UALCAN portal was used to analyze RRM2 protein expression in HCC tissues compared with normal tissues. Furthermore, immunohistochemistry (IHC) data for RRM2 protein expression in both normal and cancerous hepatocytes were retrieved from The Human Protein Atlas (HPA, http://www.proteinatlas.org/) (Sjöstedt et al. 2020).

### Construction of RRM2-targeting vectors

Two guide RNAs (gRNAs) were designed via the CRISPOR (v 4.99, http://crispor.tefor.net/) web tool (Concordet and Haeussler 2018) and validated for specificity and minimal off-target effects via the Synthego guide verification tool (https://design.synthego.com/). Each gRNA was cloned separately into the pCAG-eCas9-GFP-U6-gRNA vector (a gift from Jizhong Zou; Addgene plasmid #79145; http://n2t.net/addgene:79145; RRID: Addgene_79145) via *BbsI* cloning sites. The cloned vectors were subsequently transformed into the *HB101* competent strain, which was verified via colony PCR, followed by Sanger sequencing using the hU6-F primer as the sequencing primer (Table 2). The verified clones were amplified in LB media supplemented with ampicillin, after which the plasmids were extracted and purified via the PureLink™ Expi Endotoxin-Free Maxi Plasmid Purification Kit (Invitrogen, USA) and used for subsequent experiments.

**Table 2:** List of oligonucleotides used in this study.

Application	Name	Forward Sequence (5’ − 3’)	Reverse Sequence (5’ − 3’)
**Cloning**	gRNA1	**CACCG**GAAGATGACAAAGCGGCGG	**AAAC**CCGCCGCTTTGTTCATCTC**C**
	gRNA2	**CACCG**CGCGGCGCGGGAGATTTAA	**AAAC**TTAAATCTCCCGCGCCGCG**C**
**Colone PCR and Sanger sequencing after transformation**	hU6-F	GAGGGCCTATTTCCCATGATT	-
**PCR and sanger sequencing after transfection**	RRM2-gRNA1 loci	CCAAAGCCGCATTGTTTCCT	TCTTCTGGGGTCCTCCGATT
	RRM2-gRNA2 loci	GCTCCTCACGCAATCCTAAA	GACACGGAGGGAGAGCATAG

**PCR**: polymerase chain reaction, **gRNA**: guide RNA, **hU6-F**: human U6 promoter forward, **RRM2**: ribonucleotide reductase regulatory subunit M2

### Cell culture

HepG2 cells were obtained from the American Type Culture Collection (ATCC, https://www.atcc.org/) and cultured in Dulbecco’s modified Eagle’s medium (DMEM, Invitrogen, USA) supplemented with 10% fetal bovine serum (FBS, Invitrogen, USA) and 1% penicillin‒streptomycin (Invitrogen, USA). The cells were subsequently incubated at 37 °C in a humidified atmosphere containing 5% CO2. The cells were subcultured with trypsin-EDTA (Invitrogen, USA) when they reached 70–80% confluency.

### Transient transfection

HepG2 cells were seeded at an average density of 1.8 × 10⁴ cells/cm² the day before transfection, unless mentioned otherwise. The cells were then transiently transfected using Lipofectamine™ 3000 (Invitrogen, USA) according to the manufacturer’s protocol with either the null vector as a negative control (NC), or the gRNA1-containing vector (gRNA1), or the gRNA2-containing vector (gRNA2) or co-transfected with both the gRNA1 and gRNA2 vectors (gRNA1+2). Wild-type untransfected (UT) HepG2 cells served as a control. After transfection, the cells were incubated for 72 h before being used in subsequent experiments. To validate the transfection efficiency, green fluorescent protein (GFP), which is upstream of gRNA, was used to visualize the cells under a confocal fluorescence microscope (Model DMi8 autom, CMS GmbH; Leica Microsystems, Wetzlar, Germany). The mean fluorescence intensity (MFI) was analyzed by Leica Application Suite X (LAS X, core software v 3.7.4; Leica) in randomly captured fields to quantify GFP expression.

### Genome editing efficiency

Following transfection, genomic DNA was extracted from HepG2 cells, and then the loci surrounding each gRNA target site were PCR amplified using AmpliTaq Gold^®^ 360 Master Mix (Invitrogen, USA) according to the manufacturer’s protocol with an adjusted annealing temperature of 58 °C. The amplified products were then run on agarose gels, purified using the GeneJET Gel Extraction Kit (Thermo Scientific, USA), and subjected to Sanger sequencing via a Macrogen 3730xl DNA Analyzer. The resulting chromatograms were analyzed via the Tracking of Indels by DEcomposition (TIDE v 3.3.0, https://tide.nki.nl/) online tool and the Applied Biosystems SeqScreener Gene Edit Confirmation (SGC, https://apps.thermofisher.com/apps/gea-web/) web application. These analyses provided data on indel formation, editing efficiency, and frameshift percentages. The primer pairs used for amplification and sequencing are listed in Table 2.

### RNA extraction and quantitative RT‒PCR

Total RNA was extracted from the cells with TRIzol reagent (Invitrogen, USA) according to the manufacturer’s instructions. The concentration and purity of the RNA were evaluated with a Qubit™ 4 fluorometer (Invitrogen, USA). A RevertAid First Strand cDNA Synthesis Kit (Thermo Scientific, USA) was used for cDNA synthesis, followed by PCR amplification via Maxima SYBR Green qPCR Master Mix (2X) (Thermo Scientific, USA) on a 7500 Fast Real-Time PCR System (Applied Biosystems, USA). Relative mRNA expression levels were calculated via the 2^−ΔΔCt^ method and normalized to the level of GAPDH. The RRM2 primer pair was designed via the Primer3Plus web tool (https://www.primer3plus.com/) (Untergasser et al. 2012) with the following sequences: forward, 5′-CTGGCTCAAGAAACGAGGAC-3′ and reverse, 5′-TCAGGCAAGCAAAATCACAG-3′.

### Western blotting

Harvested cells were lysed via RIPA buffer, and equal protein concentrations of the lysates (25 µg) were separated via SDS‒PAGE and transferred onto PVDF membranes. For immunoblotting, the membranes were incubated with primary antibodies against RRM2 (Invitrogen, USA), GSS (Invitrogen, USA), GAPDH (Invitrogen, USA), or β-actin (Invitrogen, USA) at a 1:1000 dilution, followed by detection with the corresponding secondary antibodies. The secondary antibodies used were anti-mouse IgG, HRP-linked (Cell Signaling, USA), and anti-rabbit IgG, HRP-linked (Invitrogen, USA), at a dilution of 1:3000. Chemiluminescence detection was performed via Pierce ECL Western Blotting Substrate (Thermo Scientific, USA), and images were acquired with a ChemiDoc imaging system (Bio-Rad, USA). Band intensities were quantified via ImageJ software (Schneider et al. 2012) and normalized to those of β-actin or GAPDH.

### Viability assay

Cell viability was assessed via the MTT assay at 3, 4, and 5 days post transfection. At each time point, a total volume of 25 µL of MTT reagent (5 mg/mL) (Sigma‒Aldrich, USA) was added to each well of a 96-well plate and incubated for 4 h at 37 °C. Following incubation, the medium was carefully removed, and 100 µL of DMSO was added to each well and incubated for 15 min at 37 °C until complete solubilization of the formed crystals occurred. The absorbance was measured at 570 nm through a microplate reader (Bio-Rad Laboratories Inc., model 3350, Hercules, California, USA). The absorbance values, which are directly proportional to the number of viable cells, were used to calculate cell viability as a percentage relative of the UT HepG2 cells.

### Colony formation assay

The clonogenic potential of HepG2 cells post transfection was evaluated via a colony formation assay. The cells were seeded in 6-well plates at a density of 3000 cells/well and cultured for 14 days under standard conditions. After the incubation period, the colonies were rinsed with PBS, fixed with methanol and stained with crystal violet (0.5% w/v) for 30 min. Colonies were imaged and quantified through the ImageJ software colonyArea plugin (Guzmán et al. 2014).

### Scratch assay

The cell migration potential following transfection was assessed by the monolayer wound healing assay. At 72 h post transfection, when the cells had reached 90–100% confluence, a uniform scratch was made across the cell monolayer using a sterile 100 µL pipette tip. Then, the wells were washed three times with PBS to remove detached cells, and fresh medium was added. Images of the scratch were captured immediately after wounding (0 h) and at subsequent time points (24, 48 and 72 h) using a ZEISS Primovert inverted microscope with an Axiocam ERc 5s camera (Carl Zeiss Microscopy GmBh, Germany). The wound areas were quantified by ImageJ software (Schneider et al. 2012), and the percentages of wound closure over time were calculated by comparing the wound area at each time point to the initial area at 0 h.

### Cell cycle analysis

To assess the cell cycle distribution, the cells were harvested and fixed in ice-cold 60% ethanol for 1 h at 4 °C. The fixed cells were subsequently resuspended in PBS containing propidium iodide (PI) (10 µg/mL) and RNase A (50 µg/mL) and incubated in the dark for 20 min at 37 °C. PI-stained cells were analyzed for DNA content via an ACEA Novocyte™ flow cytometer (ACEA Biosciences Inc., San Diego, CA, USA) with an FL2 (λex/em 535/617 nm) detector, and cell cycle phase distributions were quantified via ACEA NovoExpress™ software.

### Apoptosis analysis

Apoptosis was assessed by staining cells with Annexin V-FITC/PI solution in the dark for 30 min at room temperature via an Annexin V-FITC apoptosis detection kit (Abcam Inc., Cambridge, UK) according to the manufacturer’s protocol. FITC and PI signals were detected via the FL1 (λex/em 488/530 nm) and FL2 (λex/em 535/617 nm) channels of the ACEA Novocyte™ flow cytometer (ACEA Biosciences Inc., San Diego, CA, USA). For each sample, 12,000 events were recorded, and cells positive for FITC and/or PI were quantified by ACEA NovoExpress™ software.

### Transmission electron microscopy

The morphology and ultrastructure of the HepG2 cells were examined via transmission electron microscopy (TEM). The cells were harvested and fixed in 2.5% glutaraldehyde in 0.1 M phosphate buffer (pH 7.4) for 4 h at 4 °C. Then, the cells were washed with phosphate buffer and postfixed in 1% osmium tetroxide for 1 h at room temperature. The samples were then dehydrated through a graded series of ethanol (40%, 60%, 70%, 80%, 90%, 95% and 100%), filtered with acetone and embedded in epoxy resin. Ultrathin sections (70 nm) were cut with a PTXL ultramicrotome (RMC, Boeckeler Instruments; USA), collected on copper grids and stained with uranyl acetate and lead citrate. Finally, the samples were examined using a TEM (JEM 1200EX II, Jeol; Japan) operated at an accelerating voltage of 80 kV.

### Statistical analysis

The experiments were performed in three independent replicates. The data were analyzed with GraphPad Prism software version 8.0.2 (GraphPad Inc., USA) and are expressed as the means ± standard deviations (SDs), unless otherwise specified. For comparisons between two groups, the two-tailed Student’s t-test was used, and for multiple comparisons, one-way ANOVA followed by Tukey’s post hoc test was employed. *P* < 0.05 was considered to indicate statistical significance.

## Results

### Identification of the common upregulated DEGs in HCC and selection of RRM2 for validation

**Fig. 1:**
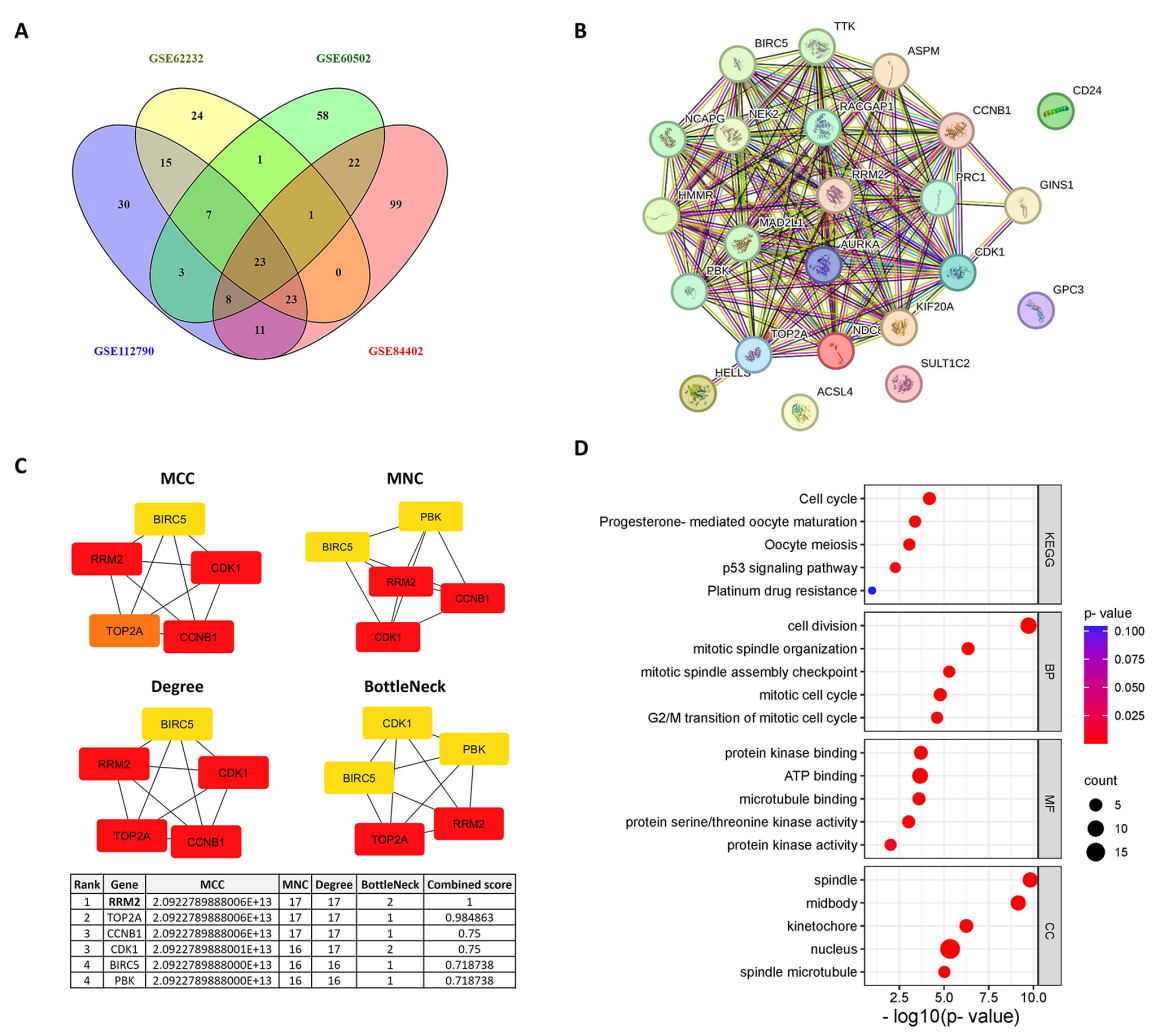
Integrated analysis of upregulated genes in tumor vs. non-tumor liver tissues. **(A)** Venn diagram showing the overlapping genes from the intersection of four independent Gene Expression Omnibus (GEO) datasets (“GSE112790”, “GSE62232”, “GSE60502” and “GSE84402”), adj-*P* value < 0.05 and log2 (FC) > 2 were set as thresholds; DEGs: Differentially expressed genes. **(B)** Protein‒protein interaction (PPI) network constructed from the 23 overlapping genes, illustrating their interactions and functional associations. The network was created via the Search Tool for the Retrieval of Interacting Genes (STRING), with a PPI enrichment *P* value < 1.0e-^16^. **(C)** Identification and ranking of hub genes on the basis of maximal clique centrality (MCC), multinode centrality (MNC), degree of connectivity, and bottleneck scores within the PPI network, as analyzed via the Cytohubba plugin in Cytoscape software. Red indicates a high score, yellow indicates a low score, and a color between red and yellow indicates a medium score. **(D)** Bubble diagrams showing the top enriched KEGG pathways and GO terms of the 23 overlapping genes ranked by − log10(*P* value); KEGG: Kyoto Encyclopedia of Genes and Genomes, GO: Gene Ontology, B: Biological Process, MF: Molecular Function, CC: Cellular Component

Our comparative analysis of tumor versus non-tumor liver tissues across the four GEO datasets revealed 120, 94, 123, and 187 upregulated DEGs in the GSE112790, GSE62232, GSE60502 and GSE84402 datasets, respectively. As illustrated in Fig. 1A, the intersection of these upregulated DEGs across the four datasets revealed 23 overlapping and consistently upregulated genes: GPC3, ASPM, RRM2, ACSL4, CDK1, TOP2A, SULT1C2, RACGAP1, HMMR, CCNB1, NDC80, GINS1, PBK, PRC, AURKA, TTK, NEK2, CD24, MAD2L1, BIRC5, NCAPG, HELLS and KIF20A. The full lists of the upregulated genes in each dataset are displayed in the supplementary table (S1).

The PPI network was constructed from these 23 overlapping genes, as shown in Fig. 1B, revealing their interactions and functional connectivity. The network included 23 nodes and 140 edges, with an average node degree of 12.2, an average local clustering coefficient of 0.808, and a PPI enrichment *P* value of < 1.0e^− 16^. Hub genes within this PPI network were identified and ranked on the basis of four centrality measures: MCC, MNC, degree of connectivity, and bottleNeck scores. Figure 1C illustrates the top five hub genes selected by CytoHubba for each of these metrics with their corresponding scores, highlighting RRM2, TOP2A, CCNB1, CDK1, BIRC5 and PBK as the top hub genes. Interestingly, RRM2 exhibited a consistently high ranking across all the metrics, suggesting its selection for subsequent validation and in vitro experiments as a potential therapeutic target for HCC.

Furthermore, functional enrichment analysis was performed to identify the top enriched KEGG pathways and GO terms associated with the 23 overlapping genes. Figure 1D highlights the most significant KEGG pathways and GO terms. The key enriched KEGG pathways included the cell cycle and P53 signaling pathways. The enriched GO terms included cell division, the mitotic spindle assembly checkpoint, the mitotic cell cycle, and the G2/M transition of mitosis for biological processes (BP); protein kinase binding, ATP binding, and microtubule binding for molecular functions (MF); and the spindle midbody, kinetochore, and nucleus for cellular components (CC).

The full lists of enriched GO terms (BP, MF and CC) and KEGG pathways are displayed in the supplementary table (S2).

### Exploring the therapeutic potential of targeting RRM2 in HCC

RRM2 (ribonucleotide reductase regulatory subunit M2), which is the key regulator subunit of ribonucleotide reductase, plays a crucial role in nucleotide metabolism by catalyzing the conversion of ribonucleotides into deoxyribonucleotides. This ensures a balanced supply of deoxyribonucleotide triphosphates (dNTPs), which are necessary for both nuclear and mitochondrial DNA biosynthesis, repair and replication. Maintaining this balance is vital for cell survival and genome stability (Kitab and Tsukiyama-Kohara 2023b; Zuo et al. 2024).

A literature review revealed that RRM2 is commonly upregulated in many cancers, including HCC (Jiang et al. 2023; Shan et al. 2022; Wang et al. 2024; Zhan et al. 2021; Zuo et al. 2024). Its overexpression leads to genome instability, oncogenic pathways activation, and, subsequently, tumor growth, cell proliferation and resistance to chemotherapy (Kitab and Tsukiyama-Kohara 2023b; Zuo et al. 2024). High RRM2 levels are also correlated with early HCC recurrence, particularly in HCV- and HBV-related HCC, as it promotes viral RNA replication (Kitab and Tsukiyama-Kohara 2023b; Tan et al. 2022; Wang et al. 2021; P.-M. Yang et al. 2020a, b). Moreover, RRM2 overexpression protects against cell death by reversing drug-induced autophagy through increasing the intracellular dNTP pool and reducing the LC3B-II level (Chen et al. 2014; P.-M. Yang et al. 2020a, b; Zuo et al. 2024), inhibiting apoptosis through the PI3K-Akt-mTOR and P53 pathways (Kitab and Tsukiyama-Kohara 2023b; Wang et al. 2021), and suppressing ferroptosis by enhancing glutathione synthesis in liver cancer cells (Tan et al. 2022; Yang et al. 2020a, b; Zhang et al. 2024; Zuo et al. 2024). It also promotes tumor immune escape (Mao et al. 2022; Qin et al. 2023), promotes angiogenesis (Kitab and Tsukiyama-Kohara 2023b) and drives communication within the tumor microenvironment (Chen et al. 2021; Zuo et al. 2024), thereby facilitating HCC progression.

**Fig. 2:**
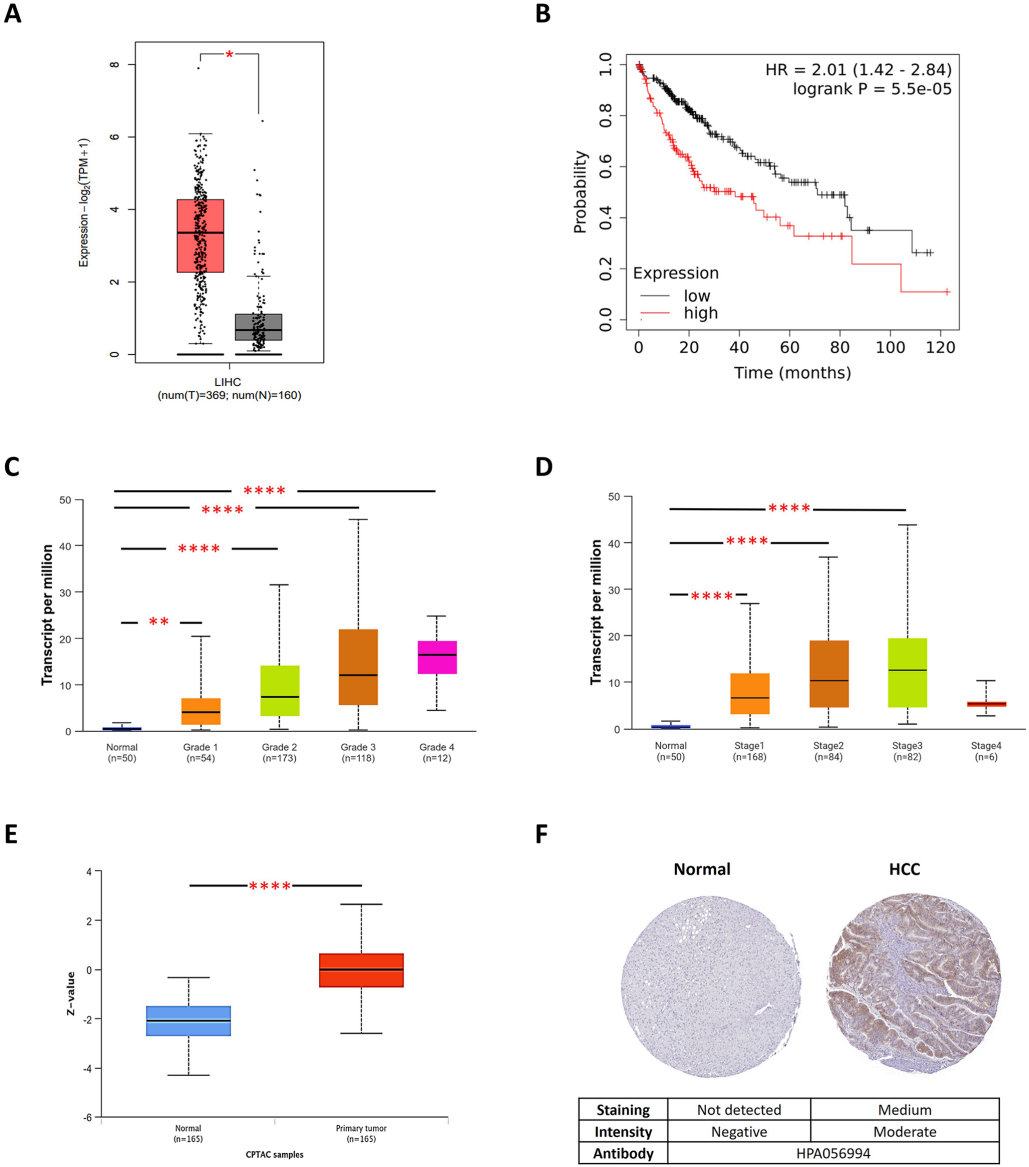
RRM2 expression and survival analysis in HCC. **(A)** mRNA expression of RRM2 in HCC (in red) and normal (in gray) liver samples analyzed via the Gene Expression Profiling Interactive Analysis 2 (GEPIA2) web tool; LIHC: Liver Hepatocellular Carcinoma. **(B)** Impact of RRM2 expression on the overall survival of HCC patients analyzed via Kaplan‒Meier Plotter. The high- and low-RRM2 expression groups are shown in red and black, respectively. *P* < 0.05 was the significance threshold, HR: hazard ratio. (**C-D)** RRM2 mRNA expression in different liver tumor grades (C) and stages (D) analyzed by The University of ALabama at the Birmingham CANcer Data Analysis Portal (UALCAN). (**E)** RRM2 protein expression in HCC (in red) and normal (in blue) liver samples analyzed via the UALCAN portal. (**F)** Representative immunohistochemistry (IHC) images of RRM2 protein expression in normal and tumor hepatocytes; images were obtained from the Human Protein Atlas. HCC: hepatocellular carcinoma, *P < 0.05, **P < 0.01, ***P < 0.001, ****P < 0.0001

Furthermore, our in silico analysis of multiple databases provided key insights into the role of RRM2 in HCC. Transcriptomic analysis revealed that RRM2 was significantly overexpressed in the LIHC group compared with the normal liver group, as shown in Fig. 2A, suggesting a potential role for RRM2 in the development or progression of HCC. Survival analysis revealed that patients with high RRM2 expression had a shorter OS, with an HR of 2.01 and a median survival of 38.3 months, than patients with low RRM2 gene expression, who had a median survival of 71 months (Fig. 2B), indicating the association of RRM2 expression with poorer prognosis in HCC patients. Additionally, RRM2 expression was significantly associated with both tumor grade and stage in LIHC (Fig. 2C-D), suggesting that RRM2 may be involved in tumor progression and aggressiveness. Proteomic data from the UALCAN portal supported the mRNA findings, revealing significantly higher protein expression of RRM2 in HCC tissues than in normal tissues (Fig. 2E). IHC analysis via the HPA revealed moderate to high RRM2 staining in 20% of liver cancer samples, which is the highest percentage among other cancer types, whereas RRM2 protein expression was undetectable in normal hepatocytes (Fig. 2F), suggesting that RRM2 is also active at the proteomic level in HCC.

### Efficient transfection and RRM2 gene editing

**Fig. 3:**
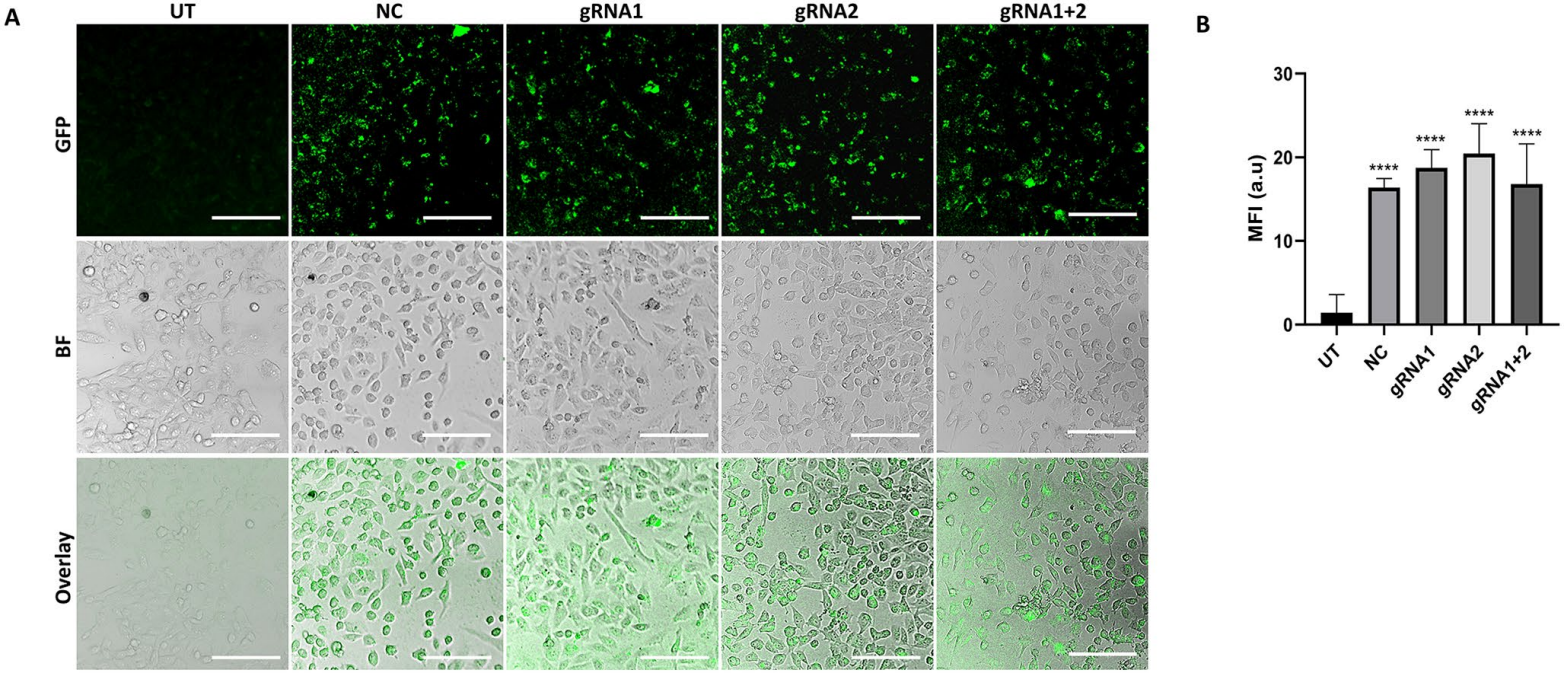
GFP expression and fluorescence intensity in HepG2 cells following transfection with RRM2-targeting vectors. **(A)** Representative confocal microscopy images showing GFP expression 72 h post transfection in untransfected (UT), null vector-transfected (NC), gRNA1 vector-transfected (gRNA1), gRNA2 vector-transfected (gRNA2) and gRNA1 & gRNA2-cotransfected (gRNA1+2) HepG2 cells. GFP: green fluorescent protein channel; BF: bright field; overlay: combined GFP and BF images. Scale bar: 100 μm. **(B)** Quantification of GFP fluorescence intensity. MFI: mean fluorescence intensity; a.u: arbitrary units; ****P < 0.0001

The success of RRM2-targeting vectors transfection was evaluated based on GFP expression. Weak GFP signals were detected in the UT cells, whereas strong GFP signals were observed in the transfected cells; representative images are shown in Fig. 3A. As demonstrated in Fig. 3B, a significant increase in MFI was observed in the transfected cells compared with the UT control cells, indicating successful transfection.

**Fig. 4:**
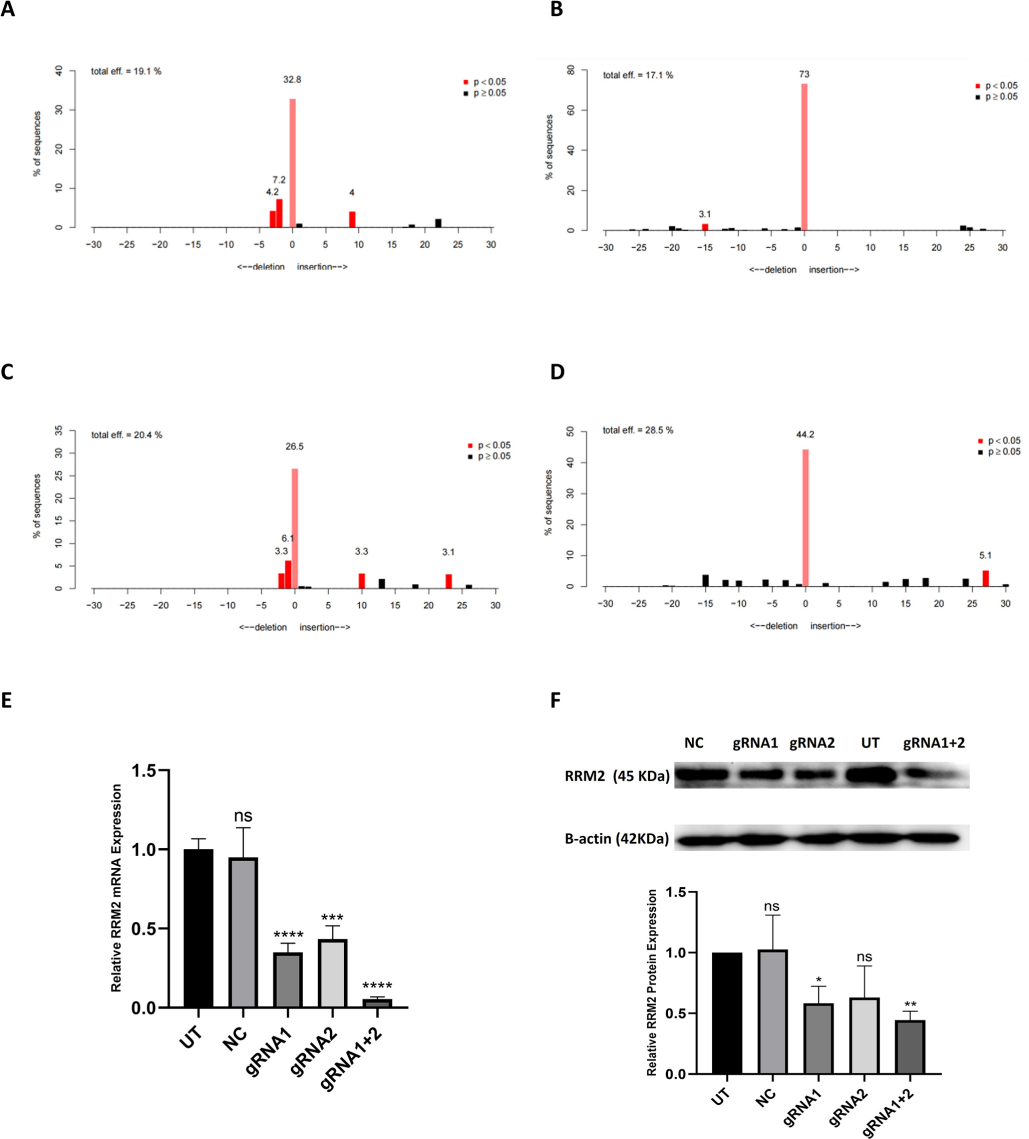
Assessment of genome editing efficiency and RRM2 expression post transfection; **(A-D)** Tracking of Indels by DEcomposition (TIDE) analyses of HepG2 mixed populations, showing indel spectra and frequencies in gRNA1 vector-transfected cells (A), gRNA2 vector-transfected cells (B), gRNA1+2 co-transfected cells at the gRNA1 locus (C) and gRNA1+2 co-transfected cells at the gRNA2 locus (D). (**E)** RRM2 mRNA expression in untransfected (UT), null vector-transfected (NC), gRNA1 vector-transfected (gRNA1), gRNA2 vector-transfected (gRNA2) and gRNA1 & gRNA2 co-transfected (gRNA1+2) HepG2 cells relative to the UT cells. (**F)** Representative Western blot images and quantification of RRM2 protein levels normalized to those of β-actin in parallel experiments with the same cells. ns: nonsignificant, *P < 0.05, **P < 0.01, ***P < 0.001, ****P < 0.0001

RRM2 gene editing efficiency following transfection was subsequently evaluated through TIDE analysis. In the gRNA1-, gRNA2-, and gRNA1+2-transfected cells, the sequencing electropherograms exhibited notable noise and disturbance in comparison with those of the UT and NC controls, indicating specific RRM2 editing events at the targeted loci. As shown in Fig. 4 (A-D), TIDE analysis revealed total editing efficiencies of 19.1%, 17.1%, 20.4% and 28.5% for gRNA1 (flanking the gRNA1 locus), gRNA2 (flanking the gRNA2 locus), gRNA1+2 (flanking the gRNA1 locus) and gRNA1+2 (flanking the gRNA2 locus)-transfected cells, respectively. The combined gRNA1+2 strategy demonstrated the highest overall editing efficiency and frameshift mutation rate, highlighting its effectiveness in disrupting RRM2.

### CRISPR/Cas9-mediated gene targeting significantly downregulates RRM2 expression in HepG2 cells

The effectiveness of CRISPR/Cas9-mediated RRM2 targeting was further investigated by analyzing RRM2 expression at both the mRNA and protein levels. The RT‒qPCR results revealed significant downregulation of RRM2 mRNA levels in the gRNA1 (0.35-fold), gRNA2 (0.43-fold), and gRNA1+2 (0.05-fold) cells compared with the UT cells (Fig. 4E). Consistent with the qPCR results, Western blotting confirmed the downregulation of RRM2 protein expression. There was a marked decrease in RRM2 protein expression in gRNA1 (0.58-fold) and gRNA2 (0.63-fold) cells, with gRNA1+2 (0.44-fold) cells showing the most significant effect (Fig. 4F). Notably, there was no significant difference between the NC and UT cells, indicating that our gRNAs specifically inhibited RRM2 expression in HCC cells.

These findings collectively demonstrate the effectiveness of our CRISPR/Cas9 targeting strategy in downregulating RRM2 at both the transcriptomic and proteomic levels. Additionally, the results highlight the enhanced efficacy of the combined gRNA1+2 strategy over the single gRNA approach in RRM2 downregulation; therefore, gRNA1+2 was chosen for subsequent experiments.

### RRM2 downregulation inhibits the viability and colony formation of HepG2 cells

**Fig. 5:**
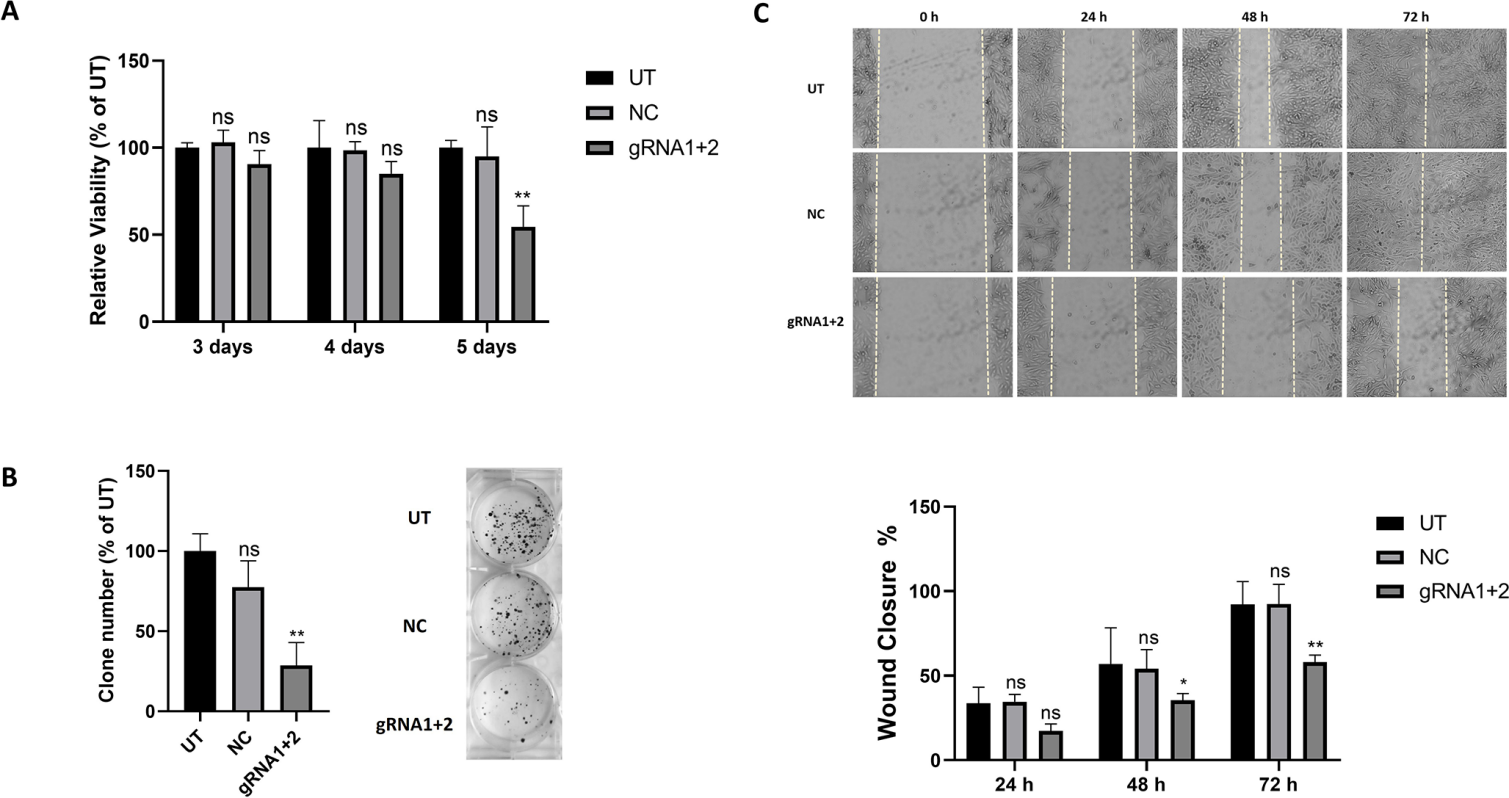
Impact of RRM2 knockdown on the viability,clonogenic potential and migration of HepG2 cells. **(A)** MTT assay: Bar graph showing HepG2 cell viability on days 3, 4 and 5 post transfection in untransfected (UT), null vector-transfected (NC) and gRNA1 & gRNA2 co-transfected (gRNA1+2) HepG2 cells as a percentage of UT cells. **(B)** Colony formation assay: representative images of the colonies formed by HepG2 cells after 2 weeks. The graph shows the number of colonies relative to the number of UT cells. **(C)** Scratch assay: representative images of the wound area (10× magnification) at 0, 24, 48 and 72 h post scratch. The graph shows the percentage of wound closure at 24, 48 and 72 h. ns: nonsignificant *P < 0.05, **P < 0.01, ***P < 0.001, ****P < 0.000

To investigate the impact of RRM2 downregulation on restoring normal cellular functions and reversing liver cancer characteristics, we assessed the viability and clonogenic potential of HepG2 cells after transfection via MTT and colony formation assays, respectively. As shown in Fig. 5A, the MTT assay results demonstrated a notable, time-dependent decrease in HepG2 cell viability in the gRNA1+2-transfected cells compared with the control cells (UT and NC), with a significant 45% reduction observed on the fifth day post transfection. These findings suggest that RRM2 plays a crucial role in maintaining HepG2 cell viability.

Moreover, the colony formation assay revealed a significant decrease in both the number and size of colonies formed, with a 71% reduction in the colony formation rate in RRM2-downregulated cells compared with UT control cells (Fig. 5B). These findings highlight the role of RRM2 in HepG2 cell proliferation and clonogenic survival.

### RRM2 downregulation impairs the migration of HepG2 cells

The wound healing scratch assay revealed significant differences in the migratory behavior of HepG2 cells following transfection. At 0 h, the initial scratch area was consistent across all the cells; however, RRM2-downregulated cells exhibited slower wound closure than control cells did (Fig. 5C). The control cells (UT and NC) achieved 100% wound closure by 72 h, whereas the gRNA1+2-transfected cells exhibited only 58% closure (Fig. 5C), suggesting that RRM2 knockdown inhibits cell migration and may impact metastasis.

### RRM2 downregulation induces cell cycle arrest and cell death in HepG2 cells

**Fig. 6:**
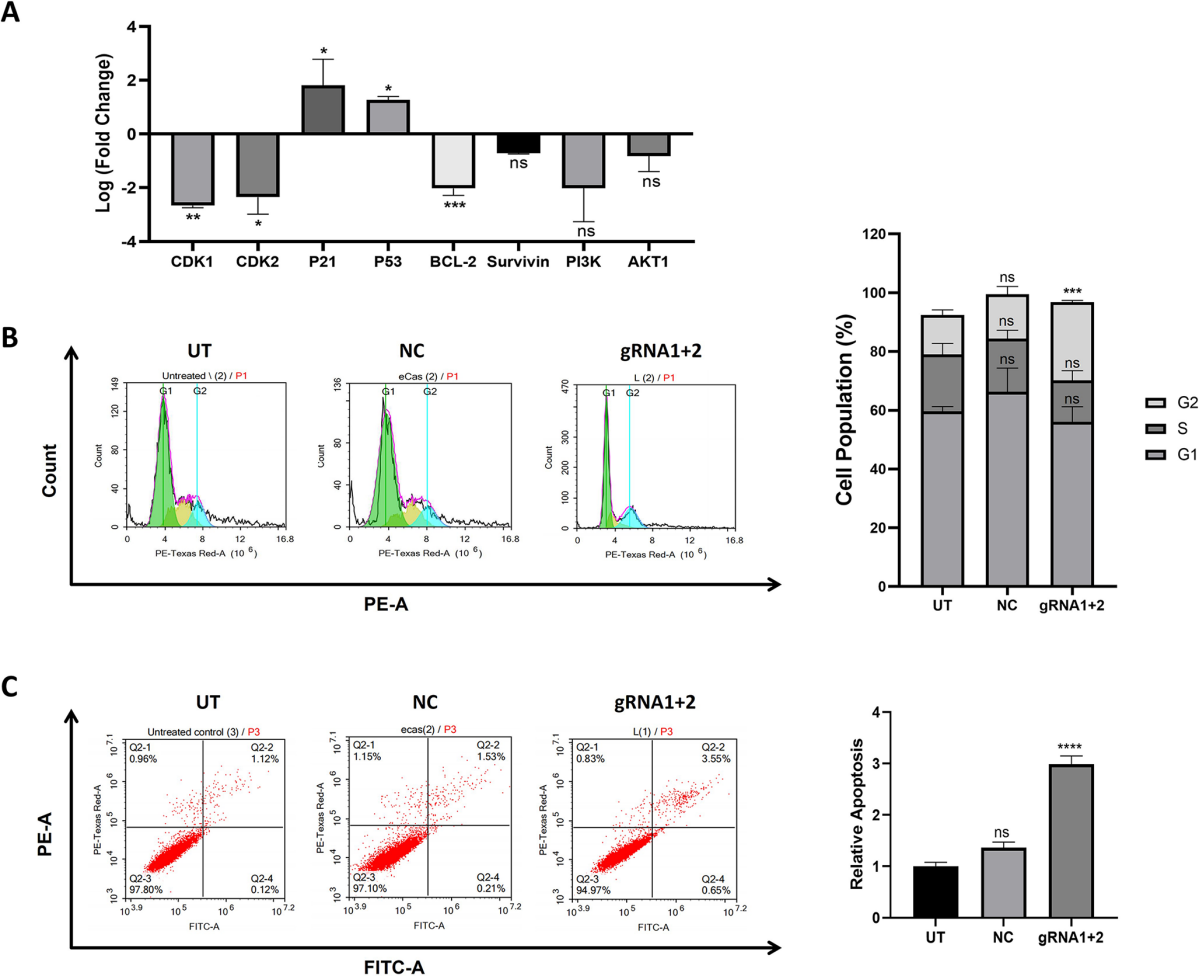
RRM2 knockdown induces cell cycle arrest and apoptosis in HepG2 cells. **(A)** RT‒qPCR analysis of selected key regulatory genes involved in the cell cycle and apoptosis. The results are presented as the log2 (fold change) change in mRNA expression for the gRNA1 & gRNA2 co-transfected (gRNA1+2) HepG2 cells relative to the null vector-transfected (NC) cells. The bar chart shows significant upregulation and downregulation. **(B)** Representative flow cytometric plots showing the cell cycle distribution analysis of untransfected (UT), NC and gRNA1+2 HepG2 cells. The bar chart represents the percentage of cells in the G1, S and G2 phases. **(C)** Representative flow cytometric plots showing the levels of apoptosis in the same cells. The bar chart illustrates the relative increase in apoptosis in the gRNA1+2 cells compared with the UT cells. ns: nonsignificant *P < 0.05, **P < 0.01, ***P < 0.001, ****P < 0.0001

To better understand the observed impact of RRM2 knockdown on HepG2 cells, further qPCR analysis was conducted. This analysis revealed considerable alterations in key genes involved in cell proliferation, the cell cycle and apoptosis regulation. As shown in Fig. 6A, the expression of the CDK1, CDK2, PI3K, AKT1, BCL-2, and Survivin genes was notably lower in the gRNA1+2-transfected cells than in the control cells. Conversely, the P53 and P21 genes were notably upregulated. These gene expression changes suggest an enhanced apoptotic response and cell cycle arrest resulting from RRM2 knockdown.

Accordingly, flow cytometry analysis was performed to explore the cell cycle distribution and percentage of apoptotic cells. As shown in Fig. 6B, a significant accumulation of cells at the G2 phase was observed in the gRNA1+2-transfected cells (27%) compared with 13.5% and 15% in the UT and NC cells, respectively. This significant G2 arrest highlights the impact of RRM2 knockdown on the disruption of DNA synthesis and damage repair mechanisms, thereby preventing progression beyond the G2/M phase and reversing cellular proliferation. Additionally, quantitative analysis of the flow cytometry data revealed a statistically significant increase in the percentage of cells undergoing apoptosis (2.9-fold) in the gRNA1+2 cells compared with the UT cells, without a significant increase in the NC control cells (Fig. 6C).

### RRM2 knockdown induces stress and morphological changes in HepG2 cells

**Fig. 7:**
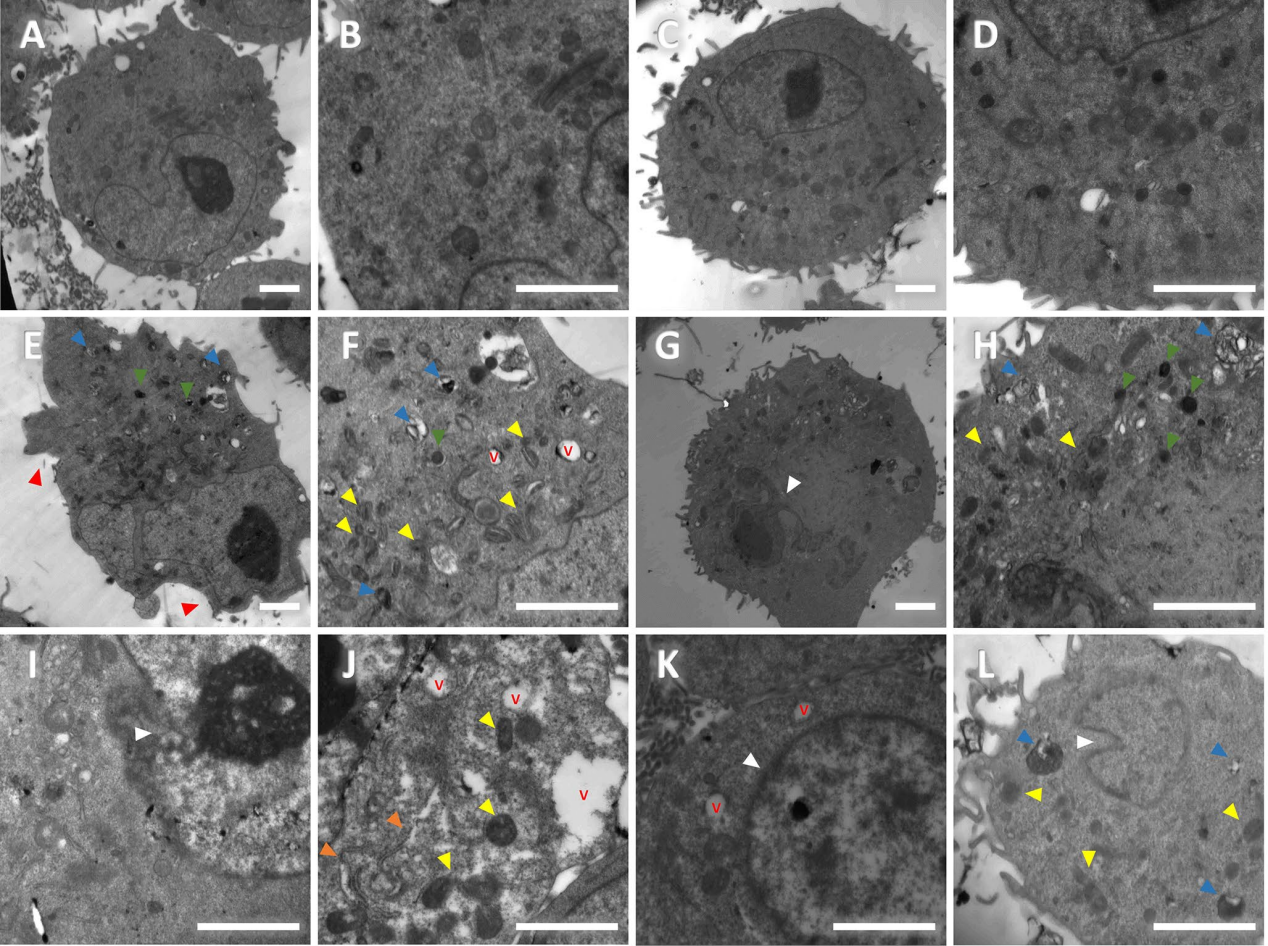
RRM2 knockdown induces morphological and ultrastructural changes in HepG2 cells. Representative transmission electron microscopy (TEM) images of untransfected (A-B), null vector-transfected (C-D) and gRNA1 & gRNA2-cotransfected (E-L) HepG2 cells. Scale bar: 2 μm, magnification: 2000x (A, C, E, G) and 5000x (B, D, F, H-L). Yellow arrowhead: mitochondria; blue arrowhead: autophagic vesicles; green arrowhead: lysosomes; red arrowhead: cell membrane; white arrowhead: nuclear membrane; orange arrowhead: swollen endoplasmic reticulum; V: vacuoles

TEM analysis revealed additional insights into the morphological and ultrastructural changes in HepG2 cells after transfection. The UT cells displayed typical HepG2 morphology, including regular cell membranes, numerous intact organelles, mitochondria with intact cristae, a euchromatic nucleus with a defined nuclear membrane and a prominent nucleolus, and fewer cytoplasmic vacuoles (Fig. 7A-B). Similarly, the NC cells showed no substantial differences from the UT cells, maintaining normal HepG2 cell morphology (Fig. 7C-D).

In contrast, HepG2 cells transfected with gRNA1+2 exhibited notable changes, indicating cellular stress. These changes included irregular or blebbed cell membranes (Fig. 7E), disrupted organelles, particularly mitochondria with damaged cristae (Fig. 7E, F, G, H, J, L), nuclear membrane blebbing (Fig. 7G) or disruption (Fig. 7I), a shifted cytoplasm (Fig. 7G), chromatin condensation (Fig. 7K), and nuclear indentations (Fig. 7L). Additionally, there was a swollen endoplasmic reticulum (Fig. 7J), increased numbers of autophagic vesicles and lysosomes (Fig. 7E-F, G-H, L), and more numerous and larger vacuoles (Fig. 7J, F). These morphological alterations suggest that RRM2 knockdown induces cell stress, which affects cell viability and function and eventually leads to cell death. To further investigate the impact of RRM2 knockdown on the observed cellular stress, the expression of glutathione synthetase (GSS), a key enzyme involved in glutathione (GSH) synthesis and antioxidant defense against ferroptosis, was analyzed via Western blotting. Compared with control cells, RRM2-knockdown cells (gRNA1+2) presented a significant reduction in GSS protein levels (Supplementary Fig. S3). This downregulation of GSS likely impaired antioxidant defense mechanisms, exacerbated oxidative stress and contributed to the observed cellular stress induced by RRM2 knockdown.

## Discussion

HCC has attracted global and local attention because of its high incidence and mortality rates, which are accompanied by poor survival outcomes and limited therapeutic options for rapidly developing resistance (Bray et al. 2024; Sankar et al. 2024; Youness et al. 2024). Recent advances in bioinformatics and the growing availability of multiomics data, such as microarray data, have transformed the landscape of cancer research and opened new avenues for identifying novel genetic and epigenetic therapeutic targets in HCC (Tsimberidou et al. 2022). Gene therapy has made significant progress in recent years, particularly with CRISPR/Cas9, which enables precise, efficient and targeted gene editing (Amjad et al. 2024). Combining gene therapy with other therapies has shown synergistic potential, overcoming the limitations of conventional therapies and positioning gene therapy as a promising standalone or synergistic component in multimodal strategies (Amjad et al. 2024; Ma et al. 2024).

In this study, we analyzed four GEO microarray datasets (GSE112790, GSE62232, GSE60502 and GSE84402) to identify the upregulated DEGs between tumor and non-tumor liver tissues. Twenty-three overlapping upregulated DEGs were identified and further analyzed for functional and pathway enrichment. These genes were found to be involved in cell cycle-related biological processes, including cell division, mitotic spindle organization, the mitotic spindle assembly checkpoint and the G2/M transition. These processes are tightly controlled in normal cells but often dysregulated in cancer cells, contributing to the aggressive proliferation observed in HCC (Greenbaum 2004; Williams and Stoeber 2012). Moreover, key pathways in the KEGG database, such as the cell cycle, p53 signaling, cellular senescence, nucleotide metabolism, apoptosis and viral carcinogenesis, were enriched, highlighting the complexity of liver cancer pathogenesis and suggesting that dysregulation of these pathways drives tumor progression and resistance to therapy. Through PPI network analysis, RRM2, TOP2A, CCNB1, CDK1, BIRC5, and PBK were identified as potential therapeutic and prognostic targets for HCC. Notably, most of these genes have been reported in previous similar studies as promising targets (Caputo et al. 2023; Chen et al. 2021; Moghimi et al. 2024; Nguyen et al. 2021); however, few experimental studies have been conducted on these genes; thus, their role in HCC is still unclear.

RRM2, a critical component of the ribonucleotide reductase enzyme essential for DNA synthesis and repair, emerged as the top candidate in our analysis across multiple metrics. Accordingly, RRM2 was selected for further validation. To confirm its selection, both a literature review and bioinformatic analysis were conducted, which revealed that RRM2 overexpression in HCC was consistently correlated with tumor growth, drug resistance, low survival rates and tumor aggressiveness (Chen et al. 2021; Kitab and Tsukiyama-Kohara 2023b; Qin et al. 2023; Tan et al. 2022; Yang et al. 2020a, b). These findings highlight the potential therapeutic benefits of targeting RRM2 to restore normal hepatocellular functions. Similarly, recent studies have identified RRM2 as a promising therapeutic target in HCC and urged its experimental validation (Caputo et al. 2023; Mao et al. 2022; Qin et al. 2023). However, the specific influence of RRM2 knockdown in HCC, particularly via the use of CRISPR/Cas9, has not been thoroughly explored. In the present study, we addressed this gap by investigating the impact of RRM2 knockdown in HepG2 cells utilizing CRISPR/Cas9 as a novel therapeutic strategy.

The efficiency of CRISPR/Cas9-mediated genome editing is widely variable and depends on many factors, including the gRNA structure, number of gRNAs used, Cas9 variant, target gene and host-related factors, delivery method and incubation period (Javaid and Choi 2021; Jung et al. 2024; Motoche-Monar et al. 2023). In this study, two gRNAs were utilized to enhance RRM2-targeting specificity and efficiency. They were designed to target the early, conserved translated exons shared across all RRM2 isoforms, thereby increasing the chance of producing frameshift mutations, in alignment with best practices outlined by Gundry et al. and others (Gerashchenkov et al. 2020; Gundry et al. 2017; Tsakirpaloglou et al. 2023). Additionally, a high-fidelity Cas9 variant (eSpCas9) was also utilized to further increase specificity (Guo et al. 2023; Javaid and Choi 2021). While the specificity of the designed gRNAs was computationally validated using the Synthego guide verification tool and CRISOR off-target score, both with high specificity and minimal off-target activity, experimental validation of potential off-target effects was not conducted in this study. This remains a limitation that should be addressed in future research. Subsequently, the targeting vectors, each containing a single gRNA, were constructed, validated and transfected into HepG2 cells, either with one vector or by co-transfecting both vectors. Our results revealed a modest knockout efficiency in the transiently transfected cell pools, with an approximately 1.6-fold increase in the co-transfected cells compared with those transfected with a single gRNA. This observation aligns with Javaid and Choi’s findings that using multiple gRNAs at lower concentrations can improve editing efficiency, likely by minimizing off-target effects while maximizing on-target gene editing (Javaid and Choi 2021). Accordingly, RRM2 expression was significantly downregulated at both the transcriptomic and proteomic levels, with even greater downregulation observed in the co-transfected cells, leading us to utilize the dual strategy for the subsequent experiments. Remarkably, to our knowledge and based on a comprehensive literature review, this is the first study to assess RRM2 editing efficiency in HepG2 cells, so our findings provide a basis for future research, suggesting the use of stable transfections and/or different delivery methods to further enhance RRM2 editing efficiency.

The knockdown of RRM2 significantly impacts cell viability, proliferation, migration, cell cycle progression and apoptosis in HepG2 cells, as demonstrated by multiple in vitro functional assays. The MTT assay revealed a marked reduction in cell viability, and the monolayer colony formation assay demonstrated significant inhibition of the clonogenic potential of HepG2 cells. These findings align with the established role of RRM2 in maintaining the dNTP pool, which is crucial for DNA synthesis, repair and replication (Kitab and Tsukiyama-Kohara 2023b; Zuo et al. 2024). Furthermore, the results of the wound healing assay revealed significant migration suppression in HepG2 cells following RRM2 knockdown. Previous studies have indicated that RRM2 promotes cell proliferation, migration and angiogenesis through the PI3K/AKT pathway and that targeting RRM2 in NSCLC and breast cancer inhibits these processes by reducing PI3K/AKT expression at both the mRNA and protein levels (Han et al. 2021; Zhuang et al. 2020). Consistent with these studies, we observed downregulation of PI3K and AKT1 mRNA levels following RRM2 knockdown in HepG2 cells, suggesting that RRM2 regulates the PI3K/AKT pathway across various cancer types, including HCC. Notably, these assays focused on the time-dependent effects of RRM2 knockdown, regardless of the transfected DNA dose. Future studies assessing the dose dependency and threshold levels of RRM2 expression required to produce the observed phenotypes would provide deeper insights.

Cell cycle and apoptosis analyses revealed that RRM2 knockdown resulted in a pronounced accumulation of HepG2 cells in the G2/M phase and a marked increase in apoptotic cells, which further contributed to the observed impairment of cell growth and proliferation. Although the exact mechanisms by which RRM2 inhibition induces cell cycle arrest and apoptosis in HCC are not yet fully understood, RRM2 regulates these processes through multiple interconnected pathways. RRM2 depletion disrupts dNTP pool homeostasis, leading to DNA replication stress and activation of the ATR/CHK1 checkpoint pathway. This activation triggers G2/M arrest and apoptosis (Koppenhafer et al. 2020; Ma et al. 2017). In addition, the replication stress induced by RRM2 downregulation triggers p53 activation, leading to the upregulation of p21 (Jin et al. 2020). p21 inhibits the cyclin A/CDK1 and cyclin B/CDK1 complexes, thereby blocking the G2/M transition and inducing cell cycle arrest. If DNA damage is irreparable, sustained p53 activation further upregulates proapoptotic targets such as Puma and Bax, triggering mitochondrial outer membrane permeabilization, caspase activation, and ultimately apoptosis (Chen 2016; Karimian et al. 2016). Furthermore, RRM2 knockdown reduces CDK1 protein levels through enhanced autophagy-dependent degradation, further contributing to G2/M arrest (Jiang et al. 2022). Moreover, RRM2 suppression results in the accumulation of reactive oxygen species (ROS) (Yang et al. 2020a, b; Zhang et al. 2024), which disrupts CDK1/cyclin B1 complex formation, inactivates cdc25B, and activates the p53/p21 axis, collectively enforcing G2/M arrest and promoting apoptosis via both mitochondrial- and ER stress-mediated pathways (Huang et al. 2021). In line with these mechanisms, Mazzu et al. demonstrated that RRM2 downregulation in prostate cancer cells induces widespread transcriptomic and phosphoproteomic changes, including the suppression of key genes enriched in the MYC, E2F and cell cycle pathways and the activation of genes related to the p53 and apoptosis pathways (Mazzu et al. 2019, 2020). Mazzu et al. validated these findings in other cancer cell lines, suggesting that RRM2 inhibition could affect a similar gene panel across multiple cancer types. Similarly, we found that RRM2 downregulation in HepG2 cells induced the expression of p53 and p21, whereas cdk1, cdk2, bcl2, survivin, PI3k, and AKT were downregulated.

Interestingly, recent reviews have reported the complex, overlapping, and context-dependent interplay between various forms of cell death—including apoptosis, ferroptosis and autophagy—in cancer in general, particularly in HCC, as these processes can influence one another and share several organelle dysfunctions and signaling pathways (Kouroumalis et al. 2023; Liu and Gu 2022; Zheng et al. 2023). In our study, TEM analysis provided further evidence of the cellular stress induced by RRM2 inhibition in HepG2 cells, revealing common features across apoptosis, ferroptosis and autophagy, such as endoplasmic reticulum stress and mitochondrial damage, along with typical characteristics of apoptosis, including blebbing of the cell and nuclear membranes, organelle disruption, cytoplasmic shifting, and chromatin condensation. These observations align with previous studies that proposed the multifaceted role of RRM2 beyond its role in apoptosis, including its involvement in ferroptosis and autophagy (Dai et al. 2021; Jiang et al. 2022; Li et al. 2024; Zhang et al. 2024). Yang et al. suggested that RRM2 protects cancer cells against ferroptosis specifically through regulating the GSS protein level (Yang et al. 2020a, b), which is consistent with the observed reduction in the GSS protein level after RRM2 downregulation in our study. These findings highlight the multifactorial role of RRM2 in HCC and emphasize the need to investigate the underlying detailed mechanisms in future studies.

Targeting RRM2 in HCC holds promising clinical potential, particularly when RRM2 is used in combination with other approaches. RRM2 overexpression is commonly associated with chemoresistance by enhancing DNA repair and survival signaling (Gao et al. 2013; Zhan et al. 2021); thus, inhibiting RRM2 could sensitize cancer cells to chemotherapy. For example, the codelivery of RRM2 siRNA with doxorubicin (Zhan et al. 2021) or adriamycin (Gao et al. 2013) has demonstrated superior tumor suppression in HCC.

In addition to chemotherapy, RRM2 inhibition may augment the activity of immunotherapies such as immune checkpoint inhibitors or tyrosine kinase inhibitors, as RRM2 overexpression is linked to immune escape and evasion in HCC (Mao et al. 2022; Qin et al. 2023). In renal cell carcinoma, RRM2 silencing enhances the antitumor activity of PD-L1 blockade and sunitinib, likely by modulating immune evasion pathways (Xiong et al. 2021). Additionally, given RRM2’s involvement in ferroptosis regulation, targeting RRM2 may increase the sensitivity of ferroptosis-inducing agents (e.g., sorafenib or erastin) by disrupting redox homeostasis and amplifying oxidative stress (Li et al. 2022). Furthermore, targeting RRM2 could complement antiviral therapies in HBV/HCV-related HCC by concurrently suppressing viral replication and tumor progression (Kitab and Tsukiyama-Kohara 2023a; Wu et al. 2024). These findings, combined with the strong safety and high specificity of RRM2 (Zhu et al. 2025), represent promising directions for future studies investigating the synergistic effects and translational potential of targeting RRM2 in HCC.

In summary, our study suggests that RRM2 is a potential therapeutic target for HCC, as its downregulation through CRISPR/Cas9 disrupts multiple signaling pathways in HepG2 cells, leading to reduced proliferation, impaired migration and induced cell cycle arrest and apoptosis. However, further research is needed to fully elucidate the molecular mechanisms involved. Expanding findings to in vivo studies and combining RRM2 knockdown with conventional therapies could improve therapeutic outcomes. Thus, targeting RRM2 could be a novel and effective therapeutic strategy for HCC.

## Conclusions

Our study identified six hub genes (RRM2, TOP2A, CCNB1, CDK1, BIRC5 and PBK) that are significantly associated with HCC and may serve as potential prognostic biomarkers or therapeutic targets in HCC. Experimental knockdown of RRM2 in HepG2 cells via CRISPR/Cas9 resulted in suppressed cell proliferation and induced cell death, suggesting that targeting RRM2 could effectively restore normal hepatocellular characteristics. These findings provide the basis for future in vivo and clinical research.

### Strengths and limitations

Moving beyond bioinformatic target identification by further integrating both in silico and in vitro methods provides a more robust approach. The multifactorial role of RRM2 in HCC was validated through multiple functional assays via the CRISPR/Cas9 technique. To our knowledge, this is the first study to utilize the CRISPR/Cas9 technique to target RRM2 in HCC, establishing a fundamental basis for future research. However, the need for detailed exploration of the molecular mechanisms involved and potential off-target effects of CRISPR/Cas9 editing has not been extensively addressed.

### Recommendations and future perspectives

Given these promising results, expanding our findings to in vivo studies is highly recommended to validate the therapeutic benefit of RRM2 knockdown in a more clinically relevant context. Additionally, combining RRM2 targeting with other promising chemomodulatory therapies (Anwar et al. 2022; Atta et al. 2023; Chiang et al. 2024; El Mesallamy et al. 2014) or existing HCC therapies may reveal a synergistic effect and enhance therapeutic outcomes. Further studies are needed to fully clarify the underlying molecular mechanisms and potential off-target effects, which is critical for translating this approach to clinical applications.

## Electronic supplementary material

Below is the link to the electronic supplementary material.

Supplementary Material 1

Supplementary Material 2

Supplementary Material 3

## Data Availability

Data is provided within the manuscript or supplementary information files.

## Abbreviations

BP: Biological Process
CC: Cellular Component
CRISPR/Cas9: Clustered Regularly Interspaced Short Palindromic Repeats/CRISPR-Associated Protein 9
DAVID: Database for Annotation, Visualization, and Integrated Discovery
DEGs: Differentially expressed genes
dNTP: Deoxyribonucleotide triphosphates
DSB: Double-Strand Break
FC: Fold change
GEO: Gene Expression Omnibus
GFP: Green fluorescent protein
GO: Gene Ontology
gRNA: Guide RNA
GSH: Glutathione
GSS: Glutathione Synthetase
HCC: Hepatocellular Carcinoma
HCV: Hepatitis C Virus
HepG2: Human hepatoma cell line 2
HPA: Human Protein Atlas
HR: Hazard ratio
IHC: Immunohistochemistry
KEGG: Kyoto Encyclopedia of Genes and Genomes
MCC: Maximum Clique Centrality
MF: Molecular function
MFI: Mean Fluorescence Intensity
MNC: Maximum neighborhood component
NC: Negative control
OS: Overall survival
PCR: Polymerase chain reaction
PPI: Protein‒protein interaction
qPCR: Quantitative Polymerase Chain Reaction
RRM2: Ribonucleotide Reductase M2
TEM: Transmission electron microscopy
TIDE: Tracking of Indels by Decomposition
UT: Untransfected
