# Supplementary material for: Unveiling the Therapeutic Potential of Targeting RRM2 in Hepatocellular Carcinoma: An Integrated In Silico and In Vitro Study
Source: Funct Integr Genomics. 2025 Jun 10;25(1):123. doi: 10.1007/s10142-025-01630-0 (PMC12152083; doi:10.1007/s10142-025-01630-0)
Supplement: Supplementary file 2 — Supplementary Material 2 [file 10142_2025_1630_MOESM2_ESM.pdf]

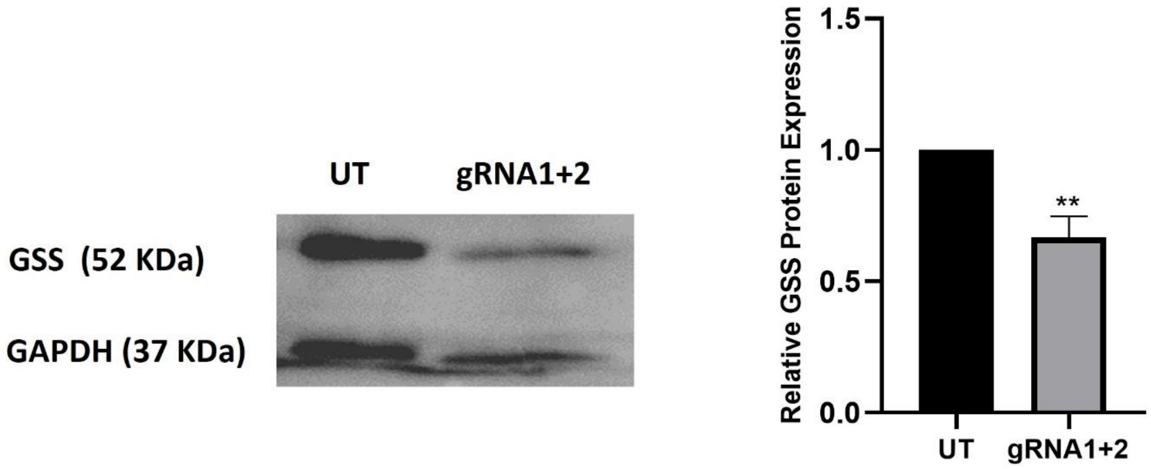

Figure (S3): Representative Western blot image of GSS and GAPDH in in the untransfected (UT) and gRNA1 & gRNA2 co-transfected (gRNA1+2) HepG2 cells. GAPDH was used for normalization. The bar chart quantifies GSS protein levels relative to the untransfected group. ns: non-significant, $**P < 0.01$.
